# Association of Autistic Traits With Depression From Childhood to Age 18 Years

**DOI:** 10.1001/jamapsychiatry.2018.1323

**Published:** 2018-06-13

**Authors:** Dheeraj Rai, Iryna Culpin, Hein Heuvelman, Cecilia M. K. Magnusson, Peter Carpenter, Hannah J. Jones, Alan M. Emond, Stanley Zammit, Jean Golding, Rebecca M. Pearson

**Affiliations:** 1 Centre for Academic Mental Health, Population Health Sciences, Bristol Medical School, University of Bristol, Bristol, United Kingdom; 2 BASS Autism Services for Adults, Avon and Wiltshire Mental Health Partnership National Health Service (NHS) Trust, Bristol, United Kingdom; 3 National Institute for Health Research (NIHR) Biomedical Research Centre, University of Bristol, Bristol, United Kingdom; 4 Department of Public Health Sciences, Karolinska Institutet, Stockholm, Sweden; 5 Centre for Epidemiology and Community Medicine, Stockholm Health Care Services, Stockholm, Sweden; 6 Medical Research Council (MRC) Integrative Epidemiology Unit, University of Bristol, United Kingdom; 7 Centre for Child and Adolescent Health, Population Health Sciences, Bristol Medical School, University of Bristol, Bristol, United Kingdom; 8 Medical Research Council (MRC) Centre for Neuropsychiatric Genetics and Genomics, Institute of Psychological Medicine and Clinical Neurosciences, Cardiff University, Cardiff, United Kingdom

## Abstract

**Importance:**

Population-based studies following trajectories of depression in autism spectrum disorders (ASD) from childhood into early adulthood are rare. The role of genetic confounding and of potential environmental intermediaries, such as bullying, in any associations is unclear.

**Objectives:**

To compare trajectories of depressive symptoms from ages 10 to 18 years for children with or without ASD and autistic traits, to assess associations between ASD and autistic traits and an *International Statistical Classification of Diseases*, *10th Revision* (*ICD-10*) depression diagnosis at age 18 years, and to explore the importance of genetic confounding and bullying.

**Design, Setting, and Participants:**

Longitudinal study of participants in the Avon Longitudinal Study of Parents and Children birth cohort in Bristol, United Kingdom, followed up through age 18 years. Data analysis was conducted from January to November 2017.

**Main Outcomes and Measures:**

Depressive symptoms were assessed using the Short Mood and Feelings Questionnaire (SMFQ) at 6 time points between ages 10 and 18 years. An *ICD-10* depression diagnosis at age 18 years was established using the Clinical Interview Schedule–Revised. Exposures were ASD diagnosis and 4 dichotomized autistic traits (social communication, coherence, repetitive behavior, and sociability). An autism polygenic risk score was derived using the Psychiatric Genomics Consortium autism discovery genome-wide association study summary data. Bullying was assessed at ages 8, 10, and 13 years.

**Results:**

The maximum sample with complete data was 6091 for the trajectory analysis (48.8% male) and 3168 for analysis of depression diagnosis at age 18 years (44.4% male). Children with ASD and autistic traits had higher average SMFQ depressive symptom scores than the general population at age 10 years (eg, for social communication 5.55 [95% CI, 5.16-5.95] vs 3.73 [95% CI, 3.61-3.85], for ASD 7.31 [95% CI, 6.22-8.40] vs 3.94 [95% CI, 3.83-4.05], remaining elevated in an upward trajectory until age 18 years (eg, for social communication 7.65 [95% CI, 6.92-8.37] vs 6.50 [95% CI, 6.29-6.71], for ASD 7.66 [95% CI, 5.96-9.35] vs 6.62 [95% CI, 6.43-6.81]). Social communication impairments were associated with depression at age 18 years (adjusted relative risk, 1.68; 95% CI, 1.05-2.70), and bullying explained a substantial proportion of this risk. There was no evidence of confounding by the autism polygenic risk score. Analysis in larger samples using multiple imputation led to similar but more precise results.

**Conclusions and Relevance:**

Children with ASD and ASD traits have higher depressive symptom scores than the general population by age 10 years, which persist to age 18 years, particularly in the context of bullying. Social communication impairments are an important autistic trait in relation to depression. Bullying, as an environmental intermediary, could be a target for interventions.

## Key Points

**Question:**

Are children with autism and autistic traits at greater risk of depression at age 18 years, and are genetic confounding and bullying important in these associations?

**Findings:**

Among 6091 participants in this longitudinal study, children with autism and autistic traits had higher depressive symptom scores than the general population at age 10 years, remaining elevated in an upward trajectory until age 18 years. Social communication impairment was associated with depression at 18 years and was substantially mediated by bullying.

**Meaning:**

Social communication impairments are an important autistic trait in relation to depression; bullying may be an environmental intermediary and a target for interventions.

## Teaser

This longitudinal study compares trajectories of depressive symptoms from ages 10 to 18 years for children with or without autism spectrum disorders and autistic traits and explores the role of genetic confounding and environmental intermediaries such as bullying.

## Introduction

Autism spectrum disorders (ASD) are characterized by impairments in reciprocal social interaction and by repetitive and stereotyped interests and behaviors.^1^ The autism spectrum is a heterogeneous construct, and its component traits are distributed across the population,^2,3^ with potentially distinct etiologies^4,5^ and outcomes. Despite increasing recognition in recent years,^6^ there are substantial gaps in our understanding of the outcomes of children with ASD as they transition into adulthood.

Depression is disabling and is common in children with ASD, but few longitudinal population-based studies have followed the natural history of depression in ASD or its component traits.^7^ Because family members of children with ASD also have an increased risk of depression,^8,9,10^ a genetic overlap between ASD and depression is possible. However, depression in family members could also relate to difficulties associated with having a child with greater needs or behavioral difficulties.

Regardless of a genetic basis, it is possible that there are modifiable factors that could be targeted by interventions to reduce the risk of depression in individuals with autism. In clinical practice, individuals with autism seen with depression often report histories of traumatic experiences, particularly bullying. Bullying is strongly associated with depression, an effect that may endure into adulthood,^11^ and could thus be important in the association between autism and depression.^12^ For instance, bullying could be a mediator on the causal pathway between autism and depression. It is also possible that the negative effect of bullying on depression may be amplified in the context of the social impairments in autism. To our knowledge, no longitudinal studies have explored these potential mechanisms.

This study used data from a large population-based cohort in England. Our objectives were to (1) compare trajectories of depressive symptoms from ages 10 to 18 years for children with or without ASD or high scores on autistic trait measures, (2) assess whether children with ASD and autistic traits were at increased risk of depression at age 18 years, (3) explore the role of genetic confounding in these associations, and (4) explore the importance of bullying in any associations.

## Methods

### Study Cohort

The Avon Longitudinal Study of Parents and Children (ALSPAC) is a birth cohort study that enrolled mothers in early pregnancy in Bristol and surrounding areas in 1990 to 1992 in England.^13,14^ It has detailed information on parents and children, collected prospectively at multiple times during pregnancy and throughout childhood. Data sources include self-report questionnaires, clinical assessments, biological samples, and birth, medical, and educational records. The study website contains details of all the data available in a fully searchable format (http://www.bris.ac.uk/alspac/researchers/data-access/data-dictionary/). We used all available data for each combination of exposure and outcome, and we imputed values for missing covariate data using multiple imputation (details are shown in eFigure 1 in the Supplement).

Ethical approval for all data collected in the ALSPAC was obtained from the ALSPAC Ethics and Law Committee and the local research ethics committees. Participants provided written informed consent for all clinic assessments, and consent was implied if questionnaires were returned. Participants were followed up through age 18 years. Data analysis was conducted from January to November 2017.

### Ascertainment of Autism and Autistic Traits

We identified children with ASD using a multisource approach, including a review of clinical records of all children who had multidisciplinary assessment for a developmental disorder (validated against *International Statistical Classification of Diseases*, *10th Revision* [*ICD-10*] criteria by a consultant pediatrician^15^), educational records of special education support provided for ASD, and parental reports of an autism or Asperger syndrome diagnosis.^16^ The ASD cases have been cross-validated against the ASD trait measures,^16,17^ and as reported below in the Results section, they are associated with a polygenic risk score (PRS) for autism.

By age 11 years, the ALSPAC had collected 93 measures related to autistic features.^18^ Of these, the following 4 individual measures were the strongest predictors of ASD^18^: the Social Communication Disorders Checklist (SCDC) at 7 years, the coherence subscale of the Children’s Communication Checklist at 9 years, a repetitive behavior scale at 5 years, and the sociability subscale of the Emotionality Activity and Sociability temperament measure at 3 years.^16^ To define high-risk groups for these autistic traits, we dichotomized individuals closest to the worst 10% of distributions of each ASD trait measure.^17^

### Ascertainment of Depression and Depressive Traits

The Short Mood and Feelings Questionnaire (SMFQ),^19^ designed to measure depressive symptoms in children and adolescents, was administered at 6 time points between ages 10 and 18 years via postal questionnaires or in clinics. It has 13 items relating to low mood during the past 2 weeks, each with scores of 0 to 2. Individual item scores were summed, producing a 0 to 26 score range.^20^

The computerized version of the Clinical Interview Schedule–Revised (CIS-R)^21^ is a fully structured psychiatric interview widely used in community samples. It was administered at age 18 years to identify individuals with an *ICD-10* diagnosis of depression.

### Potential Confounders

We included the following variables in our models: (1) child sex, (2) parity (≤1 child vs ≥2 children), (3) maternal occupational class (manual vs nonmanual), (4) mother’s highest educational attainment, (5) financial problems (occurrence vs nonoccurrence of major financial problems), (6) maternal age at delivery (in years), (7) maternal Crown-Crisp anxiety score at 18 weeks’ gestation and 8 weeks after delivery,^22^ (8) maternal antenatal (18 and 32 weeks’ gestation) and postnatal (8 weeks and 8 months) depression measured with the Edinburgh Postnatal Depression Scale (EPDS score ≥13),^23^ and (9) accommodation type (detached house vs semidetached house vs flat). We included these variables because they are associated with both autism and depression, apart from being predictors of attrition in the ALSPAC.

### Bullying in Late Childhood and Early Adolescence

Relational and overt bullying was assessed as separate yes or no items at ages 8, 10, and 13 years using the modified Bullying and Friendship Interview Schedule.^24^ We created a latent construct of bullying based on 6 binary measures (relational and overt bullying assessed at ages 8, 10, and 13 years) using factor analysis to identify the common variance in the items. Conceptually, this latent construct represents the tendency of children to be bullied persistently throughout childhood or adolescence and was used for the mediation analysis described below in the Statistical Analysis subsection. We also created a binary variable to capture no vs any overt or relational bullying at any time, which we used for testing interactions described below in the Statistical Analysis subsection.

### PRS for Autism

We examined potential genetic confounding of associations between ASD and depression using autism PRSs, calculated for genotyped ALSPAC children using summary data from the Psychiatric Genomics Consortium autism discovery genome-wide association study (GWAS) (eMethods 1 in the Supplement).^25^ We created a set of scores based on single-nucleotide polymorphisms (SNPs) that were associated with an ASD diagnosis at a range of GWAS *P* value thresholds (.5 to 1e^−7^) and used PRSs generated using SNPs meeting a 0.05 GWAS *P* value threshold in our main analysis because it maximally captured autism liability within our sample (eFigure 2 in the Supplement).

### Statistical Analysis

We conducted analyses using Stata/MP (version 14; StataCorp) and Mplus (version 8; Muthén & Muthén). We examined trajectories of depressive symptoms (continuous SMFQ scores) between ages 10 and 18 years among those with or without ASD and each autistic trait using mixed-effects linear growth models. To accommodate individual differences in trends of depressive symptoms with age, we included random effects for intercept and slope coefficients and added quadratic and cubic terms to accommodate potential nonlinear trends.

We then used modified Poisson regression to estimate the relative risk (RR) of an *ICD-10* depression diagnosis at age 18 years in individuals with ASD and each autistic trait vs those without, with robust 95% CIs.^26^ We estimated crude risks, followed by adjustment for all potential confounders. We further adjusted these models for the autism PRS in the sample with genetic data.

We used path analysis to assess mediation of associations between autistic traits and depression at age 18 years by the experience of being bullied in late childhood or early adolescence using latent constructs of bullying and depression. Details are provided in eMethods 2 and eMethods 3 in the Supplement.

Finally, to separate the association of ASD diagnosis or traits with depression within and outside the context of bullying, we created categories representing 4 groups by the presence or absence of ASD or ASD traits and the presence or absence of any experiencing of bullying; we compared trajectories of depressive traits between ages 10 and 18 years using mixed-effects linear growth models as described above in the Statistical Analysis subsection. To statistically test moderating associations of bullying, we compared models that included the ASD and bullying variables with those that included only the ASD variable; we then compared models that included the statistical interaction between the ASD and bullying variables with models that included only the main effects of these variables using likelihood ratio tests.

### Missing Data

Missing data in our trajectory and age 18 years analysis are listed in eTable 4 in the Supplement. We imputed missing data for covariates and outcome using multiple imputation (eFigure 1, eMethods 4 in the Supplement). The availability of extensive auxiliary socioeconomic and clinical data (including 7 measures of depression between ages 10 and 18 years) enabled us to account for factors that may explain attrition, providing support to the missing-at-random assumption.^27^ We repeated our analyses, estimating average associations across 100 imputed data sets and calculated standard errors using the rule by Rubin.^28^

## Results

The maximum sample available with complete data on exposures, outcomes, and covariates was 6091 for the trajectory analysis (48.8% male) and 3168 for analysis of depression diagnosis at age 18 years (44.4% male) (eFigure 1 in the Supplement). The characteristics of our study sample by the presence of ASD and autistic traits are listed in eTable 1 in the Supplement (with an abridged version in Table 1). Mothers of children scoring highest on all autistic trait measures except sociability had a greater prevalence of screening positive for depression and had higher mean anxiety scores in pregnancy and the early postnatal period than the general population, although this pattern was not observed in children with ASD. Children with ASD and those scoring highest on all the autistic traits had a higher prevalence of depressive symptoms at age 10 years, a pattern that was also observed at other time points, albeit inconsistently (eTable 2 in the Supplement). Children with ASD and those scoring highest on the autistic trait measures had a consistently greater prevalence of overt and relational bullying at ages 8, 10, and 13 years than the comparison population, although the statistical evidence for such differences varied (eTable 3 in the Supplement).

**Table 1.** Characteristics of the Cohort by Exposure Status^a^

Variable	Diagnosed ASD (n = 8087)^b^			Social Communication Difficulties (n = 5954)^c^		
	No	Yes	*P* Value	No^d^	Yes^e^	*P* Value
No. (%)	7991 (98.8)	96 (1.2)	NA	5408 (90.8)	546 (9.2)	NA
Male sex, No. (%)	4083 (51.1)	79 (82.3)	<.001	2680 (49.6)	367 (67.2)	<.001
Parity ≤1, No. (%)	6540 (81.8)	83 (86.5)	.24	4511 (83.4)	436 (79.9)	.03
Maternal nonmanual occupational class, No. (%)	4305 (53.9)	65 (67.7)	.007	3131 (57.9)	280 (51.3)	.003
Mother’s university degree attainment, No. (%)	1187 (14.9)	20 (20.8)	.10	937 (17.3)	83 (15.2)	.21
Maternal EPDS score ≥12 in pregnancy, No. (%)	1040 (13.0)	14 (14.6)	.64	600 (11.1)	115 (21.1)	<.001
Maternal EPDS score ≥12 after birth, No. (%)	1056 (13.2)	11 (11.5)	.61	607 (11.2)	131 (24.0)	<.001
Financial problems since pregnancy, No. (%)	858 (10.7)	9 (9.4)	.67	503 (9.3)	74 (13.6)	.001
Maternal age at delivery, mean (SD), y	28.0 (4.5)	29.4 (4.2)	.004	28.5 (4.4)	28.1 (4.5)	.02
Maternal Crown-Crisp antenatal anxiety score, mean (SD)	4.7 (3.4)	4.8 (3.3)	.90	4.5 (3.3)	5.5 (3.7)	<.001
Maternal Crown-Crisp postnatal anxiety score, mean (SD)	3.3 (3.2)	3.2 (3.0)	.72	3.1 (3.1)	4.4 (3.7)	<.001

Abbreviations: ASD, autism spectrum disorders; EPDS, Edinburgh Postnatal Depression Scale; NA, not applicable.

^a^ A more detailed version of this table is available in eTable 1 in the Supplement. *P* values for No. (%) are by Pearson χ^2^ test. *P* values for mean (SD) are by 2-sided *t* test.

^b^ Estimates based on 8087 observations with complete data on covariates and diagnosed autism.

^c^ Estimates based on 5954 observations with complete data on covariates and the Social Communication Disorders Checklist scores.

^d^ Child has score in the lower 90 percentiles.

^e^ Child has score in the upper decile.

The autism PRS was associated with the ASD diagnosis and with measures of social communication and repetitive behavior (eFigure 2 in the Supplement), while being in the top decile of the autism PRS was associated with ASD and all 4 autism trait measures, with the exception of coherence (eFigure 3 in the Supplement). Results were generally consistent when using autism PRSs generated using SNP inclusion *P* value thresholds exceeding .001. There was no evidence of associations between the autism PRS and depression or bullying variables (eFigure 4 and eFigure 5 in the Supplement).

**Figure 1.**
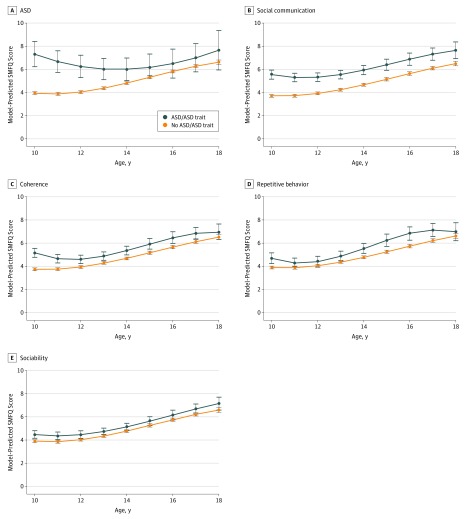
Trajectories of Depressive Symptoms in Individuals With Autism Spectrum Disorders (ASD) and ASD Traits and the Comparison Population Shown are confounder-adjusted mean Short Mood and Feelings Questionnaire (SMFQ) scores between ages 10 and 18 years among those with or without ASD and ASD traits. Fitted means were calculated using xtmixed (Stata/MP, version 14; StataCorp) multilevel regression models with linear, quadratic, and cubic terms for time. Trajectories were adjusted for child sex, parity, maternal occupational class, mother’s highest educational attainment, financial problems, maternal age at delivery, maternal Crown-Crisp anxiety score at 18 weeks’ gestation and 8 weeks after delivery, maternal antenatal (18 and 32 weeks’ gestation) and postnatal (8 weeks and 8 months) depression measured with the Edinburgh Postnatal Depression Scale, and accommodation type. Error bars indicate 95% CIs. A, ASD estimates based on 6091 observations with complete data on autism diagnosis and covariates. B, Social communication estimates based on 5209 observations with complete data on the Social Communication Disorders Checklist scores and covariates. C, Coherence estimates based on 5204 observations with complete data on coherence scores and covariates. D, Repetitive behavior estimates based on 5299 observations with complete data on repetitive behavior scores and covariates. E, Sociability estimates based on 5677 observations with complete data on sociability scores and covariates.

Examining trajectories, children with ASD and autistic traits had higher average SMFQ depressive symptom scores than the general population at age 10 years (eg, for social communication 5.55 [95% CI, 5.16-5.95] vs 3.73 [95% CI, 3.61-3.85], for ASD 7.31 [95% CI, 6.22-8.40] vs 3.94 [95% CI, 3.83-4.05], remaining elevated in an upward trajectory until age 18 years (eg, for social communication 7.65 [95% CI, 6.92-8.37] vs 6.50 [95% CI, 6.29-6.71], for ASD 7.66 [95% CI, 5.96-9.35] vs 6.62 [95% CI, 6.43-6.81]) (Figure 1). Most pronounced were differences between those with or without social communication difficulties. Analyses using imputed data sets led to similar but more precise estimates (eFigure 6 in the Supplement).

Children with social communication impairments at age 7 years were at increased risk of a diagnosis of depression at age 18 years (adjusted RR, 1.68; 95% CI, 1.05-2.70). These associations were almost unchanged after adjustment for the autism PRS (Table 2) and were estimated with greater precision in the sample without genetic data (eTable 5 in the Supplement) and after multiple imputation (eTable 6 in the Supplement). No evidence of an association between ASD and a depression diagnosis at age 18 years was observed, although the 95% CIs were wide in our main (adjusted RR, 0.50; 95% CI, 0.08-3.38) and imputed (adjusted RR, 0.80; 95% CI, 0.23-2.81) analyses.

**Table 2.** Risk of Outcome of Diagnosed Depression at Age 18 Years Among Children With Autism or Autistic Traits, Including Adjustment for Autism Polygenic Risk

Exposure	No.^a^	Crude Estimates		Adjusted Estimates^b^		With Additional Adjustment for Autism Polygenic Risk^c^	
		RR (95% CI)	*P* Value	RR (95% CI)	*P* Value	RR (95% CI)	*P* Value
ASD	2463	0.47 (0.07-3.24)	.44	0.55 (0.09-3.50)	.53	0.55 (0.09-3.49)	.53
Social communication impairments	2230	1.60 (1.00-2.54)	.048	1.68 (1.05-2.70)	.03	1.70 (1.06-2.72)	.03
Reduced speech coherence	2233	0.73 (0.38-1.41)	.35	0.72 (0.37-1.37)	.32	0.72 (0.38-1.38)	.32
Repetitive behavior	2235	1.17 (0.65-2.10)	.61	1.11 (0.61-2.00)	.74	1.11 (0.61-2.00)	.74
Reduced sociability temperament	2342	0.77 (0.45-1.31)	.34	0.84 (0.50-1.42)	.52	0.84 (0.50-1.42)	.52

Abbreviations: ASD, autism spectrum disorders; RR, relative risk (estimates were calculated using modified Poisson regression).

^a^ Number of observations with complete data on exposure, covariates, diagnosis of depression at age 18 years, and autism polygenic risk scores.

^b^ Estimates were adjusted for child sex, parity, maternal occupational class, mother’s highest educational attainment, financial problems, maternal age at delivery, maternal Crown-Crisp anxiety score at 18 weeks’ gestation and 8 weeks after delivery, maternal antenatal (18 and 32 weeks’ gestation) and postnatal (8 weeks and 8 months) depression measured with the Edinburgh Postnatal Depression Scale, and accommodation type.

^c^ Autism polygenic risk scores based on single-nucleotide polymorphisms associated with ASD at *P* < .05 in the discovery sample.

**Figure 2.**
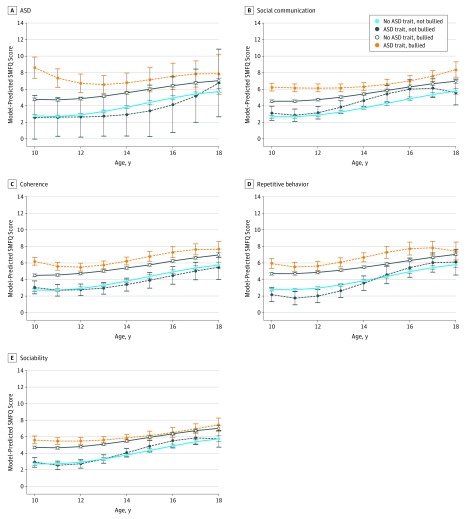
Trajectories of Depressive Symptoms in Children With or Without Autism Spectrum Disorders (ASD) and ASD Traits Within and Outside the Context of Bullying Shown are confounder-adjusted mean Short Mood and Feelings Questionnaire (SMFQ) scores among children with or without ASD and ASD traits and exposed or unexposed to bullying. Fitted means were calculated using xtmixed (Stata/MP, version 14; StataCorp) multilevel regression models with linear, quadratic, and cubic terms for time. Trajectories were adjusted for the same variables as those listed above for Figure 1. Error bars indicate 95% CIs. A, ASD estimates based on 4516 observations with complete data on autism diagnosis, bullying variables, and covariates. B, Social communication estimates based on 4041 observations with complete data on the Social Communication Disorders Checklist scores, bullying variables, and covariates. C, Coherence estimates based on 4070 observations with complete data on coherence scores, bullying variables, and covariates. D, Repetitive behavior estimates based on 4051 observations with complete data on repetitive behavior scores, bullying variables, and covariates. E, Sociability estimates based on 4268 observations with complete data on sociability scores, bullying variables, and covariates.

Children with ASD and autistic traits who also reported being bullied had the highest depression symptom scores at age 10 years, which remained elevated throughout adolescence (Figure 2). There was statistical evidence that the model that included the ASD and bullying variables explained the data better than one that included only ASD diagnosis (likelihood ratio χ^2^ = 454.75, *P* < .001) and that models with interaction terms for ASD and bullying explained the data better than models that included only the main effects of these variables (likelihood ratio χ^2^ = 5.71, *P* = .017). These different trajectories were most apparent for children with social communication difficulties and were least apparent for worst scores on sociability temperament. In the absence of bullying, the depressive symptom trajectories of children with or without ASD or autistic traits appeared broadly similar. Analyses using imputed data sets led to similar results (eFigure 7 in the Supplement).

Finally, we assessed for mediation of associations between social communication difficulties at 7 years and diagnosed depression at age 18 years by the experience of being bullied in late childhood and early adolescence (Table 3). Both before and after adjustment for potential confounders, there was strong evidence of an indirect pathway leading from social communication difficulties in early childhood to being bullied in late childhood or early adolescence to a depression diagnosis at age 18 years. We estimated that this indirect association accounted for 50.5% (95% CI, 5.5%-95.5%) of the total association of social communication difficulties with risk of depression after accounting for potential confounders. Furthermore, there was no evidence of a direct association of social communication difficulties with depression risk after accounting for the indirect association via bullying. Repeating these analyses using imputed data led to similar results (eTable 7 in the Supplement), with a more precise estimate of the indirect association accounted for by bullying (31.5%; 95% CI, 17.3%-45.7%).

**Table 3.** Association Between Social Communication Impairments at Age 7 Years and a Depression Diagnosis at Age 18 Years, Mediated by the Experience of Being Bullied in Late Childhood or Early Adolescence^a^^,^^b^

Structural Parameter Estimates	β (SE)	*P* Value	β (SE)	*P* Value
Association of exposure with mediator	0.205 (0.067)	.002	0.195 (0.070)	.005
Association of mediator with outcome	0.490 (0.089)	<.001	0.509 (0.103)	<.001
Association of exposure with outcome	0.054 (0.084)	.523	0.097 (0.087)	.268
Indirect association	0.101 (0.042)	.016	0.099 (0.045)	.026
Total association	0.155 (0.076)	.040	0.196 (0.080)	.014
Proportion of total association mediated, %	65.2	NA	50.5	NA

Abbreviations: β, unstandardized regression coefficient; CFI, Confirmatory Fit Index; NA, not applicable; RMSEA, root-mean-square error of approximation; TLI, Tucker-Lewis Index.

^a^ Depression was captured as a latent construct by means of 4 continuous measures of fatigue, concentration, sleep symptom score, and depressive symptoms. The experience of being bullied was captured as a latent construct by means of 6 binary scores capturing the child’s relational or overt status at ages 8, 10, and 13 years.

^b^ The model fit statistics for the unadjusted association (2152 observations) were RMSEA = 0.046, CFI = 0.937, and TLI = 0.918. The model fit statistics for the adjusted association (2152 observations) were RMSEA = 0.038, CFI = 0.924, and TLI = 0.903. Exposure-mediator and mediator-outcome associations were adjusted for child sex, mother’s highest educational attainment, maternal Crown-Crisp anxiety score at 18 weeks’ gestation and 8 weeks after delivery, maternal antenatal (18 and 32 weeks’ gestation) and postnatal (8 weeks and 8 months) depression measured with the Edinburgh Postnatal Depression Scale, and accommodation type.

## Discussion

In this detailed longitudinal study, we found that children with ASD and those with higher scores on all autistic trait measures had more depressive symptoms at age 10 years than the general population, and these remained elevated in an upward trajectory until age 18 years. Social communication impairments had the strongest association with a depression diagnosis at age 18 years. Findings were robust to adjustment for a range of confounders, including maternal depression and anxiety and the child’s polygenic risk for autism. We found evidence of a substantial role of bullying in contributing to and explaining a higher risk of depression in individuals with ASD and autistic symptoms.

Previous reports on this topic have been contradictory, with results of some studies^29,30^ suggesting an improvement in depressive symptoms in children with autism over time and other findings suggesting worsening,^31^ possibly because of selected and heterogeneous populations and different methods. The lack of a general population group in previous studies makes it difficult to conclude whether the trajectories of depressive symptoms in the autistic population differ from those of the general population,^29,30,31,32^ a limitation in the literature that our study attempted to address. Our findings suggesting that difficulties in social communication may have stronger associations with future depression than other autistic traits have also been reported for outcomes of suicidal thoughts and behaviors^33^ and are consistent with the concept of fractionation of component features of the autism spectrum.^4^ However, although social communication difficulties are an important feature of autism, they may occur independently in the population or within the context of other psychiatric diagnoses. Therefore, the association between social communication difficulties and depression may be important within and outside the context of ASD.

We report a significant contribution of bullying as a potential environmental intermediary between childhood autistic features and later depression. Previous work has shown strong links between the experience of bullying and later depression^34,35^; although confounding could have a role,^36^ the association is considered to be at least partially causal.^11^ In our study, children with social communication impairments were more likely to report being bullied, and the mediation analysis suggests that this explained a substantial proportion of the variance of depression at age 18 years, possibly due to reduced self-esteem or social isolation after the bullying. The risk of depression in children with greater autistic symptoms may also be amplified in the context of bullying because of preexisting underlying vulnerabilities in children with autistic features, such as impaired social skills and decreased ability to adapt to adverse or stressful events, such as being bullied. This could explain the elevated trajectories of depressive symptoms in children with ASD and autistic traits who reported being bullied. In the absence of bullying, these children appeared to follow trajectories of depressive symptoms similar to those of the general population. However, such interactions could simply suggest that bullying and autism sometimes co-occur in causal models of depression, as might be expected for any outcome of multifactorial etiology.^37^

Although it is impossible to identify the exact nature of the underlying mechanisms, our results highlight the need for further research on the role of bullying in this association and the potential for preventive interventions. Furthermore, other relevant characteristics, including comorbidities with neurodevelopmental conditions (eg, attention-deficit/hyperactivity disorder) and classroom placement could be important in this association within or outside the context of bullying and warrant future study.

The main strengths of this study were the population-based design with prospectively collected data and repeated measures of depressive symptoms, reducing the possibility of selection and recall bias and allowing us to model longitudinal trajectories. The rich covariate information enabled us to minimize the possibility of confounding bias.

### Limitations

This study has limitations. Like all cohort studies, there was significant attrition, and we used multiple imputation to limit any potential bias; however, selection bias related to missing data remains a possibility. While the use of PRSs was an advantage, they only capture common variation and were based on a small GWAS, so genetic confounding in the associations is still possible. We had insufficient numbers with an ASD diagnosis also meeting the diagnostic criteria for depression at 18 years, possibly due to selective attrition of individuals with autism with more severe depressive symptoms. This is likely to have led to the imprecise result because of a lack of statistical power. Furthermore, atypical presentations of depression are common in ASD, and our study has the potential for outcome measurement error because we used scales (eg, the CIS-R) that have not been adapted for autism.^38^ Individuals with ASD may also have difficulties in expressing and communicating their emotions and may not have sufficient verbal skills to express changes in their mood or feelings.^38^

## Conclusions

Our results suggest that ASD and autistic traits are associated with higher depressive symptom scores by age 10 years, which persist to age 18 years, particularly in the context of bullying. Social communication impairments are important in relation to a later diagnosis of depression, and bullying in adolescence could have an important role in this association. These findings add to the evidence highlighting a higher burden of depression, and also suggest a potentially modifiable pathway, through bullying. However, gaps remain in our understanding of the measurement and phenomenology of depression in individuals with autism, which could be a priority for future research. Further work could also focus on improvements in psychological^39^ and pharmacological^40^ management of depression in ASD. Finally, further research into the role of traumatic experiences, such as bullying, and the utility of interventions to reduce bullying or address its adverse effects could have the potential to reduce the burden of depression in this population.

## Notes

Supplement. **eMethods 1.** Supplemental Methods 1 **eMethods 2.** Supplemental Methods 2 **eMethods 3.** Supplemental Methods 3 **eMethods 4.** Supplemental Methods 4 **eTable 1.** Characteristics of the Cohort by Exposure Status **eTable 2.** Prevalence of Depression by Exposure Status **eTable 3.** Prevalence of Bullying Victimization by Exposure Status **eTable 4.** Characteristics of Those Complete or Missing on Covariates and Depression Diagnosis or SMFQ Scores **eTable 5.** Risk of a Depression Diagnosis at Age 18 Years Among Children With Autism or Autistic Traits **eTable 6.** Risk of Adulthood Depression Among Children With Autism or Autistic Traits (Missing Covariate and Outcome Data Predicted With Multiple Imputation) **eTable 7.** Association Between SCDC at Age 7 and a Depression Diagnosis at Age 18, Mediated by the Experience of Being Bullied in Late Childhood/Early Adolescence (Missing Covariate and Outcome Data Predicted With Multiple Imputation) **eFigure 1.** Flow Chart for Analysis Plan **eFigure 2.** Associations Between Continuous Polygenic Risk Score (PRS) for Autism and Autism Diagnosis and Autistic Traits **eFigure 3.** Associations Between Dichotomized Measure of the Autism PRS (Top Decile Versus Bottom 90 Percentiles) and Autism Diagnosis and Autistic Traits **eFigure 4.** Associations Between Continuous Polygenic Risk Score (PRS) for Autism and Diagnosed Depression at Age 18 Years (n=3,378) **eFigure 5.** Associations Between Continuous Polygenic Risk Score (PRS) for Autism and Bullying/Victimization at Any Time Point During Adolescence (n=5,032) **eFigure 6.** Confounder-Adjusted Mean MFQ Scores Between Ages 10 and 18 (Missing Covariate and Outcome Data Predicted With Multiple Imputation) **eFigure 7.** Confounder-Adjusted Mean MFQ Scores Among Children With/Without Autism or Autistic Traits and Exposed/Unexposed to Bullying (Missing Covariate and Outcome Data Predicted With Multiple Imputation)
