# Supplementary material for: Association of Autistic Traits With Depression From Childhood to Age 18 Years
Source: JAMA Psychiatry. 2018 Jun 13;75(8):835–43. doi: 10.1001/jamapsychiatry.2018.1323 (PMC6143081; doi:10.1001/jamapsychiatry.2018.1323)
Supplement: Supplement. — eMethods 1. Supplemental Methods 1 eMethods 2. Supplemental Methods 2 eMethods 3. Supplemental Methods 3 eMethods 4. Supplemental Methods 4 eTable 1. Characteristics of the Cohort by Exposure Status eTable 2. Prevalence of Depression by Exposure Status eTable 3. Prevalence of Bullying Victimization by Exposure Status eTable 4. Characteristics of Those Complete or Missing on Covariates and Depression Diagnosis or SMFQ Scores eTable 5. Risk of a Depression Diagnosis at Age 18 Years Among Children With Autism or Autistic Traits eTable 6. Risk of Adulthood Depression Among Children With Autism or Autistic Traits (Missing Covariate and Outcome Data Predicted With Multiple Imputation) eTable 7. Association Between SCDC at Age 7 and a Depression Diagnosis at Age 18, Mediated by the Experience of Being Bullied in Late Childhood/Early Adolescence (Missing Covariate and Outcome Data Predicted With Multiple Imputation) eFigure 1. Flow Chart for Analysis Plan eFigure 2. Associations Between Continuous Polygenic Risk Score (PRS) for Autism and Autism Diagnosis and Autistic Traits eFigure 3. Associations Between Dichotomized Measure of the Autism PRS (Top Decile Versus Bottom 90 Percentiles) and Autism Diagnosis and Autistic Traits eFigure 4. Associations Between Continuous Polygenic Risk Score (PRS) for Autism and Diagnosed Depression at Age 18 Years (n=3,378) eFigure 5. Associations Between Continuous Polygenic Risk Score (PRS) for Autism and Bullying/Victimization at Any Time Point During Adolescence (n=5,032) eFigure 6. Confounder-Adjusted Mean MFQ Scores Between Ages 10 and 18 (Missing Covariate and Outcome Data Predicted With Multiple Imputation) eFigure 7. Confounder-Adjusted Mean MFQ Scores Among Children With/Without Autism or Autistic Traits and Exposed/Unexposed to Bullying (Missing Covariate and Outcome Data Predicted With Multiple Imputation) [file jamapsychiatry-75-835-s001.pdf]

Supplementary Online Content

Rai D, Culpin I, Heuvelman H, et al. Association of autistic traits with depression from childhood to age 18 years. *JAMA Psychiatry*. Published online June 13, 2018. doi:10.1001/jamapsychiatry.2018.1323

eMethods 1. Supplemental Methods 1

eMethods 2. Supplemental Methods 2

eMethods 3. Supplemental Methods 3

eMethods 4. Supplemental Methods 4

eTable 1. Characteristics of the Cohort by Exposure Status

eTable 2. Prevalence of Depression by Exposure Status

eTable 3. Prevalence of Bullying Victimization by Exposure Status

eTable 4. Characteristics of Those Complete or Missing on Covariates and Depression Diagnosis or SMFQ Scores

eTable 5. Risk of a Depression Diagnosis at Age 18 Years Among Children With Autism or Autistic Traits

eTable 6. Risk of Adulthood Depression Among Children With Autism or Autistic Traits (Missing Covariate and Outcome Data Predicted With Multiple Imputation)

eTable 7. Association Between SCDC at Age 7 and a Depression Diagnosis at Age 18, Mediated by the Experience of Being Bullied in Late Childhood/Early Adolescence (Missing Covariate and Outcome Data Predicted With Multiple Imputation)

eFigure 1. Flow Chart for Analysis Plan

eFigure 2. Associations Between Continuous Polygenic Risk Score (PRS) for Autism and Autism Diagnosis and Autistic Traits

eFigure 3. Associations Between Dichotomized Measure of the Autism PRS (Top Decile Versus Bottom 90 Percentiles) and Autism Diagnosis and Autistic Traits

eFigure 4. Associations Between Continuous Polygenic Risk Score (PRS) for Autism and Diagnosed Depression at Age 18 Years (n=3,378)

eFigure 5. Associations Between Continuous Polygenic Risk Score (PRS) for Autism and Bullying/Victimization at Any Time Point During Adolescence (n=5,032)

eFigure 6. Confounder-Adjusted Mean MFQ Scores Between Ages 10 and 18 (Missing Covariate and Outcome Data Predicted With Multiple Imputation)

eFigure 7. Confounder-Adjusted Mean MFQ Scores Among Children With/Without Autism or Autistic Traits and Exposed/Unexposed to Bullying (Missing Covariate and Outcome Data Predicted With Multiple Imputation)

This supplementary material has been provided by the authors to give readers additional information about their work.

© 2018 Rai D, et al. *JAMA Psychiatry*

eMethods 1. Supplemental Methods 1

Genetic Data

Genetic data were acquired using the Illumina HumanHap550 quad genome-wide single nucleotide polymorphism (SNP) genotyping platform from 9,912 participants. Individuals were excluded from further analysis based on gender mismatches, minimal or excessive heterozygosity, disproportionate levels of individual missingness (>3%), evidence of cryptic relatedness (>10% of alleles identical by descent), and being of non-European ancestry (assessed by multidimensional scaling analysis including HapMap 2 individuals). SNPs with a minor allele frequency (MAF) of < 1%, Impute2 information quality metric of < 0.8, a call rate of < 95% or evidence for violations of Hardy-Weinberg equilibrium (P-value < $5e^{-7}$) were removed. Imputation of the target data was performed using Impute V2.2.2 against the 1000 genomes reference panel (Phase 1, Version 3; all polymorphic SNPs excluding singletons), using 2186 reference haplotypes (including non-Europeans). Following quality control assessment and imputation and restricting to 1 young person per family, genetic data was available for 8,252 ALSPAC individuals.

Polygenic Risk Score for Autism

Polygenic risk scores (PRSs) for autism were constructed, as described previously,[1, 2] using GWAS summary statistics from Psychiatric Genomics Consortium autism (PGC-ASD) discovery genome-wide association study (GWAS) on 5,305 individuals.[3] Scores were derived by summing the number of risk alleles present for each single nucleotide polymorphism (SNP) (0, 1, or 2) multiplied by the logarithm of its odds ratio for autism from the PGC-ASD GWAS. SNPs were excluded from the analysis if they had a minor allele frequency less than 0.01, imputation quality less than 0.8, or if there was allelic mismatch between samples (alleles reported by the PGC not matching alleles in the ALSPAC sample). Remaining SNPs were then pruned for LD using the PLINK (v1.90)[4] 'clump' command to retain SNPs with a trait association P-value ≤ 0.5 and $r^2 < 0.25$ within 500kb windows. Polygenic scores were calculated for each ALSPAC individual using PLINK (v1.07)[4] by summing the number of risk alleles for each SNP (0, 1 or 2) weighted by the logarithm of thr PGC-ASD GWAS odds ratio (OR). Our primary analysis used a score generated from a list of SNPs with a PGC-ASD GWAS P-value threshold ≤ 0.05. As the composition of this score will contain true and null effects, sensitivity analyses were also conducted using PRSs generated using SNPs meeting a range of P-value thresholds: ≤ 0.5 - $\leq 1e^{-7}$, allowing us to investigate the robustness of our primary analysis findings.

eMethods 2. Supplemental Methods 2

Latent Constructs and Model Fit

For mediation models, depression was measured as a latent construct, using four categorical observed measures (scoring range 0-5) capturing fatigue, sleeping patterns, concentration and depressive symptoms as factor indicators. Using chi-square, RMSEA, CFI and TLI fit statistics, we separately assessed the fit of measurement models for the bullying and depression constructs, before assessing the overall fit of a model including the structural relationships between exposure, mediator and outcome (Figure e1). Fit statistics for the bullying measurement model suggested a lack of fit to the observed data (Chi-square [df, p]=188.04 [9, <0.001]; RMSEA=0.096; CFI=0.798; TLI=0.663), while the measurement model for depression showed exact fit (chi-square [df, p]=0.68 [2, 0.71]; RMSEA=0.000; CFI=1.000; TLI=1.002). The fit of the model including structural relationships between the exposure, mediator and outcome variables was adequate (chi-square [df, p]=231.06 [42, <0.001]; RMSEA=0.046; CFI=0.937; TLI=0.918).

© 2018 Rai D, et al. *JAMA Psychiatry*

eMethods 3. Supplemental Methods 3

Mediation Effects

We examined the extent to which the association between social communication difficulties at age 7 years and a latent construct of diagnosed depression at age 18 years were statistically mediated by the experience of being bullied in late childhood and early adolescence using the mediation approach recommended by Muthén[5] to assess mediation within the context of potential confounding. We used structural equation modelling (SEM) with latent constructs of bullying and depression, and adjusted our models for a range of individual, maternal, and socioeconomic confounders modelled as binary indicators. In line with causal mediation framework[6], we assumed (i) no unmeasured confounding of exposure-outcome and mediator-outcomes associations; (ii) no mediator-outcome confounder affected by exposure; and (iii) no further intermediate confounding. We derived new parameters and standard errors representing casually-defined direct and indirect (mediated) effects[7] from estimated model parameters using the 'Model Constraint' command. Detailed description and MPlus syntax is presented in Muthén.[5] A weighted least-squares estimator was used because of its robustness in estimating both continuous and categorical variables.[8] Indirect effect and associated 95% CIs were estimated using bias-corrected bootstrapping (1,000 replications) to account for the non-normal distribution of the indirect effect.[9]

© 2018 Rai D, et al. *JAMA Psychiatry*

eMethods 4. Supplemental Methods 4

Multiple Imputation for Missing Data

We used Multivariable Imputation by Chained Equations (MICE)[10] to impute missing data in covariates and outcome. In these models, we assumed that covariate and outcome data were Missing-at-Random (MAR)[11] conditional on the variables included in our main analysis models, rich auxiliary variables capturing demographic and socioeconomic characteristics, and a wide range of detailed clinical measures of maternal and child psychopathology. We used Stata's *ice* command (version 14/MP) to generate 100 imputed datasets by 10 cycles of regression for each of the associations tested in this study, which was deemed sufficient based on the largest fraction of missing information,[12] and used the *mi estimate* prefix to estimate average associations across these 100 imputed datasets. Standard errors for associations were estimated using Rubin's rule.[13] Standard errors and 95% Confidence Intervals (CIs) for direct and indirect effects in mediation analyses of the imputed dataset were calculated using a bootstrapped estimate of 1000 samples.[14]

eReferences

1. Jones HJ, Stergiakouli E, Tansey KE, Hubbard L, Heron J, Cannon M *et al.* Phenotypic manifestation of genetic risk for schizophrenia during adolescence in the general population. *JAMA Psychiatry*. 2016; 73**:**221-228.
2. Purcell SM, Wray NR, Stone JL, Visscher PM, O'Donovan MC, Sullivan PF *et al.* Common polygenic variation contributes to risk of schizophrenia and bipolar disorder. *Nature.* 2009;460:748-752.
3. Cross-Disorder Group of the Psychiatric Genomics C, Lee SH, Ripke S, Neale BM, Faraone SV, Purcell SM *et al.* Genetic relationship between five psychiatric disorders estimated from genome-wide SNPs. *Nat Genet.* 2013;45(9)**:**984-994.
4. Purcell S, Neale B, Todd-Brown K, Thomas L, Ferreira MA, Bender D *et al.* PLINK: a tool set for whole-genome association and population-based linkage analyses. *American Journal of Human Genetics.* 2007;81:559-575.
5. Muthén BO. Applications of causally defined direct and indirect effects in mediation analysis using SEM in Mplus. *Unpublished manuscript*; 2011.
6. VanderWeele, T. Explanation in causal inference: methods for mediation and interaction. Oxford University Press; 2015.
7. Robins JM, Greenland S. Identifiability and exchangeability for direct and indirect effects. *Epidemiology*. 1992;3:143-55.
8. Muthén LK, Muthén BO, 7th ed. Mplus user's guide. Log Angeles, CA: Muthén. & Muthén, 2012.
9. MacKinnon DP, Lockwood CM, Williams J. Confidence limits for the indirect effect: distribution of the product and resampling methods. Multivar Behav Res. 2004;39:99-128.

© 2018 Rai D, et al. *JAMA Psychiatry*

eTable 1. Characteristics of the Cohort by Exposure Status

	Exposure status:	Diagnosed autism [a]			Social Communication [b]			Coherence [c]			Repetitive behaviours [d]			Sociability [e]		
		No	Yes		No [f]	Yes [g]		No [f]	Yes [g]		No [f]	Yes [g]		No [f]	Yes [g]	
	N	7,991	96		5,408	546		5,205	526		5,858	419		6,327	801	
	(%)	(98.8)	(1.2)		(90.8)	(9.2)		(90.8)	(9.2)		(93.3)	(6.7)		(88.8)	(11.2)	
		n (%)	n (%)	p [h]	n (%)	n (%)	p [h]	n (%)	n (%)	p [h]	n (%)	n (%)	p [h]	n (%)	n (%)	p [h]
Male sex		4,083 (51.1)	79 (82.3)	<0.001	2,680 (49.6)	367 (67.2)	<0.001	2,552 (49.0)	349 (66.3)	<0.001	2,962 (50.6)	262 (62.5)	<0.001	3,207 (50.7)	472 (58.9)	<0.001
Parity ≤1		6,540 (81.8)	83 (86.5)	0.24	4,511 (83.4)	436 (80.0)	0.034	4,340 (83.4)	424 (80.6)	0.11	4,854 (82.9)	340 (81.1)	0.37	5,217 (82.5)	639 (79.8)	0.062
Maternal non-manual social class	*Non-manual*	4,305 (53.9)	65 (67.7)	0.007	3,131 (57.9)	280 (51.3)	0.003	3,008 (57.8)	289 (54.9)	0.21	3,306 (56.4)	247 (58.9)	0.32	3,531 (55.8)	403 (50.3)	0.003
Maternal education	*Degree*	1,187 (14.9)	20 (20.8)	0.10	937 (17.3)	83 (15.2)	0.21	908 (17.4)	82 (15.6)	0.28	956 (16.3)	73 (17.4)	0.56	990 (15.6)	109 (13.6)	0.13
Type of dwelling	*Detached*	1,466 (18.3)	*Censor*		1,087 (20.1)	93 (17.0)		1,027 (19.7)	102 (19.4)		1,132 (19.3)	71 (16.9)		1,205 (19.0)	130 (16.2)	
	Semi-detached/terraced	5,628 (70.4)	*Censor*		3,832 (70.9)	386 (70.7)		3,717 (71.4)	363 (69.0)		4,169 (71.2)	294 (70.2)		4,489 (70.9)	577 (72.0)	
	Flat	897 (11.2)	*Censor*	0.067	489 (9.0)	67 (12.3)	0.019	461 (8.9)	61 (11.6)	0.11	557 (9.5)	54 (12.9)	0.055	633 (10.0)	94 (11.7)	0.074
Maternal EPDS ≥12 in pregnancy		1,040 (13.0)	14 (14.6)	0.64	600 (11.1)	115 (21.1)	<0.001	597 (11.5)	80 (15.2)	0.005	686 (11.7)	74 (17.7)	<0.001	796 (12.6)	106 (13.2)	0.58
Maternal EPDS ≥12 postnatally		1,056 (13.2)	11 (11.5)	0.61	607 (11.2)	131 (24.0)	<0.001	598 (11.5)	93 (17.7)	<0.001	697 (11.9)	75 (17.7)	<0.001	822 (13.0)	90 (11.2)	0.16
Major financial problems since pregnancy		858 (10.7)	9 (9.4)	0.67	503 (9.3)	74 (13.6)	0.001	480 (9.2)	70 (13.3)	0.002	559 (9.5)	66 (15.8)	<0.001	643 (10.2)	90 (11.2)	0.35

© 2018 Rai D, et al. *JAMA Psychiatry*

eTable 1: continued

	Mean (SD)	Mean (SD)	p [i]	Mean (SD)	Mean (SD)	p [i]	Mean (SD)	Mean (SD)	p [i]	Mean (SD)	Mean (SD)	p [i]	Mean (SD)	Mean (SD)	p [i]
Maternal age at delivery	28.0 (4.5)	29.4 (4.2)	0.004	28.5 (4.4)	28.1 (4.5)	0.016	28.5 (4.4)	28.7 (4.3)	0.33	28.4 (4.4)	28.6 (4.5)	0.34	28.3 (4.4)	28.0 (4.5)	0.19
Maternal Crown-Crisp antenatal anxiety	4.7 (3.4)	4.8 (3.3)	0.91	4.5 (3.3)	5.5 (3.7)	<0.001	4.5 (3.3)	5.1 (3.6)	<0.001	4.5 (3.4)	5.6 (3.5)	<0.001	4.6 (3.4)	4.7 (3.5)	0.38
Maternal Crown-Crisp postnatal anxiety	3.3 (3.2)	3.2 (3.0)	0.72	3.1 (3.1)	4.4 (3.7)	<0.001	3.1 (3.1)	4.0 (3.5)	<0.001	3.2 (3.1)	4.0 (3.5)	<0.001	3.2 (3.2)	3.3 (3.2)	0.57

Notes: (a) Estimates based on n=8,087 observations with complete data on covariates and diagnosed autism. **(b)** Estimates based on n=5,954 observations with complete data on covariates and SCDC scores. **(c)** Estimates based on n=5,731 observations with complete data on covariates and coherence scores. **(d)** Estimates based on n=6,277 observations with complete data on covariates and repetitive behaviour scores. **(e)** Estimates based on n=7,128 observations with complete data on covariates and sociability scores. **(f)** Child has score in the lower ≈90 percentiles. **(g)** Child has score in the upper ≈decile. **(h)** P-value for Pearson's Chi-square. **(i)** P-value for two-sided T-test. **(j)** *Censor* – censored to prevent disclosure due to small cell counts.

© 2018 Rai D, et al. *JAMA Psychiatry*

eTable 2. Prevalence of Depression by Exposure Status

**Exposure status:**		Diagnosed autism			Social Communication			Coherence			Repetitive behaviours			Sociability		
		No	Yes		No [a]	Yes [b]		No [a]	Yes [b]		No [a]	Yes [b]		No [a]	Yes [b]	
**Depressed mood**		n (%)	n (%)	p [c]	n (%)	n (%)	p [c]	n (%)	n (%)	p [c]	n (%)	n (%)	p [c]	n (%)	n (%)	p [c]
MFQ ≥10 at age 10 [j]	No	4,642 (92.2)	31 (64.6)		3,838 (93.4)	302 (80.8)		3,908 (93.4)	315 (84.9)		3,936 (92.6)	231 (85.9)		3,925 (92.6)	481 (88.1)	
	Yes	392 (7.8)	17 (35.4)	<0.001	271 (6.6)	72 (19.3)	<0.001	277 (6.6)	56 (15.1)	<0.001	315 (7.4)	38 (14.1)	<0.00 1	316 (7.5)	65 (11.9)	<0.001
MFQ ≥10 at age 12.5 [j]	No	4,189 (91.4)	38 (74.5)		3,479 (91.8)	295 (85.5)		3,503 (91.9)	307 (87.2)		3,588 (91.7)	217 (86.1)		3,538 (91.4)	451 (90.0)	
	Yes	394 (8.6)	13 (25.5)	<0.001	310 (8.2)	50 (14.5)	<0.001	309 (8.1)	45 (12.8)	0.003	324 (8.3)	35 (13.9)	0.002	331 (8.6)	50 (10.0)	0.29
MFQ ≥10 at age 13.5 [j]	No	3,597 (85.9)	39 (79.6)		3,011 (86.5)	239 (78.4)		3,015 (86.4)	274 (83.8)		3,086 (86.0)	190 (81.2)		3,056 (86.1)	395 (84.0)	
	Yes	592 (14.1)	10 (20.4)	0.21	472 (13.6)	66 (21.6)	<0.001	476 (13.6)	53 (16.2)	0.20	504 (14.0)	44 (18.8)	0.044	494 (13.9)	75 (16.0)	0.23
MFQ ≥10 at age 16.5 [j]	No	2,827 (79.7)	39 (79.6)		2,457 (81.0)	176 (75.9)		2,437 (80.7)	201 (77.3)		2,479 (80.6)	153 (73.2)		2,450 (80.0)	294 (79.7)	
	Yes	719 (20.3)	10 (20.4)	0.98	577 (19.0)	56 (24.1)	0.057	583 (19.3)	59 (22.7)	0.19	597 (19.4)	56 (26.8)	0.010	614 (20.0)	(75) 20.3	0.90
MFQ ≥10 at age 17 [j]	No	2,373 (75.8)	24 (64.9)		2,016 (76.7)	136 (65.7)		2,007 (76.9)	155 (68.3)		2,047 (76.6)	126 (66.7)		2,035 (76.2)	247 (73.3)	
	Yes	756 (24.1)	13 (35.1)	0.12	612 (23.3)	71 (34.3)	<0.001	604 (23.1)	72 (31.7)	0.004	625 (23.4)	63 (33.3)	0.002	637 (23.8)	90 (26.7)	0.25
MFQ ≥10 at age 18 [j]	No	1,817 (76.)	24 (75.0)		1,561 (76.4)	115 (71.4)		1,560 (76.4)	127 (74.7)		1,584 (76.1)	105 (72.4)		1,560 (76.1)	201 (74.4)	
	Yes	573 (24.0)	8 (25.0)	0.89	482 (23.6)	46 (28.6)	0.15	482 (23.6)	43 (25.3)	0.62	497 (23.9)	40 (27.6)	0.31	490 (23.9)	69 (25.6)	0.55

© 2018 Rai D, et al. *JAMA Psychiatry*

eTable 2: continued

Exposure status:		Diagnosed autism			Social Communication			Coherence			Repetitive behaviours			Sociability		
		No	Yes		No [a]	Yes [b]		No [a]	Yes [b]		No [a]	Yes [b]		No [a]	Yes [b]	
**Depression diagnosis**		n (%)	n (%)	p [c]	n (%)	n (%)	p [c]	n (%)	n (%)	p [c]	n (%)	n (%)	p [c]	n (%)	n (%)	p [c]
Child diagnosed with depression at age 18 [k]	No	*Cens*	*Cens*		2,439 (93.1)	187 (89.5)		2,434 (92.7)	204 (94.4)		2,490 (93.0)	161 (89.9)		2,472 (92.5)	308 (93.6)	
	Yes	*Cens*	*Cens*	0.34	182 (6.9)	22 (10.5)	0.054	191 (7.3)	12 (5.6)	0.35	188 (7.0)	18 (10.1)	0.13	200 (7.5)	21 (6.4)	0.47

Notes: (a) Child has score in the lower ≈90 percentiles. **(b)** Child has score in the upper ≈decile. **(c)** P-value for Pearson's Chi-square. **(d)** *Cens* – censored to prevent disclosure due to small cell counts.

© 2018 Rai D, et al. *JAMA Psychiatry*

eTable 3. Prevalence of Bullying Victimization by Exposure Status

**Exposure status:**		Diagnosed autism			Social Communication			Coherence			Repetitive behaviours			Sociability		
		No	Yes		No [a]	Yes [b]		No [a]	Yes [b]		No [a]	Yes [b]		No [a]	Yes [b]	
**Bullying**		n (%)	n (%)	p [c]	n (%)	n (%)	p [c]	n (%)	n (%)	p [c]	n (%)	n (%)	p [c]	n (%)	n (%)	p [c]
Victim of overt bullying at age 8	No	3,321 (67.1)	23 (53.5)		2,795 (68.7)	201 (53.3)		2,790 (68.7)	196 (54.3)		2,831 (67.8)	167 (61.4)		2,802 (67.1)	348 (66.4)	
	Yes	1,627 (32.9)	20 (46.5)	0.058	1,271 (31.3)	176 (46.7)	<0.001	1,271 (31.3)	165 (45.7)	<0.001	1,347 (32.2)	105 (38.6)	0.030	1,372 (32.9)	176 (33.6)	0.74
Victim of relational bullying at age 8	No	4,109 (84.9)	32 (84.2)		3,409 (85.5)	297 (83.0)		3,412 (85.5)	274 (80.6)		3,489 (85.3)	215 (82.4)		3,476 (84.9)	429 (84.6)	
	Yes	733 (15.1)	6 (15.8)	0.91	578 (14.5)	61 (17.0)	0.19	580 (14.5)	66 (19.4)	0.015	602 (14.7)	46 (17.6)	0.20	617 (15.1)	78 (15.4)	0.85
Victim of overt bullying at age 10	No	3,961 (78.8)	31 (70.5)		3,306 (80.5)	240 (65.8)		3,348 (80.2)	258 (71.1)		3,359 (79.2)	200 (75.8)		3,354 (79.2)	413 (76.3)	
	Yes	1,068 (21.2)	13 (29.6)	0.18	800 (19.5)	125 (34.3)	<0.001	828 (19.8)	105 (28.9)	<0.001	883 (20.8)	64 (24.2)	0.19	882 (20.8)	128 (23.7)	0.13
Victim of relational bullying at age 10	No	4,588 (92.3)	38 (88.4)		3,780 (92.9)	316 (88.0)		3,846 (92.9)	316 (89.0)		3,879 (93.5)	239 (91.2)		3,872 (92.3)	491 (92.3)	
	Yes	384 (7.7)	5 (11.6)	0.34	287 (7.1)	43 (12.0)	0.001	294 (7.1)	39 (11.0)	0.007	317 (7.6)	23 (8.8)	0.47	321 (7.7)	41 (7.7)	0.97
Victim of overt bullying at age 13	No	3,486 (77.3)	35 (68.6)		2,936 (78.6)	230 (67.3)		2,942 (78.2)	257 (73.0)		2,994 (77.7)	182 (71.9)		2,950 (77.3)	380 (77.3)	
	Yes	1,025 (22.7)	16 (31.4)	0.14	798 (21.4)	112 (32.8)	<0.001	820 (21.8)	95 (27.0)	0.025	859 (22.3)	71 (28.1)	0.034	869 (22.8)	112 (22.8)	>0.99
Victim of relational bullying at age 13	No	4,142 (92.2)	42 (82.4)		3,448 (92.7)	304 (89.7)		3,480 (92.7)	308 (89.8)		3,564 (92.8)	220 (88.4)		3,524 (92.6)	434 (89.1)	
	Yes	350 (7.8)	9 (17.7)	0.009	273 (7.3)	35 (10.3)	0.047	276 (7.4)	35 (10.2)	0.056	278 (7.2)	29 (11.7)	0.010	281 (7.4)	53 (10.9)	0.007

Notes: (a) Child has score in the lower ≈90 percentiles. **(b)** Child has score in the upper ≈decile. **(c)** P-value for Pearson's Chi-square**.**

© 2018 Rai D, et al. *JAMA Psychiatry*

eTable 4. Characteristics of Those Complete or Missing on Covariates and Depression Diagnosis or SMFQ Scores

		Sample used for depression at age 18 analysis			Sample used for age 10-18 years trajectory analysis		
		Complete	Missing		Complete	Missing	
		n (%)	n (%)	P-value Chi-square test	n (%)	n (%)	p
Male sex	Male	1,405 (44.4)	6,116 (53.2)		2,972 (48.8)	1,433 (47.0)	
	Female	1,763 (55.7)	5,377 (46.8)	<0.001	3,119 (51.2)	1,617 (53.0)	0.10
Parity ≤1	0 or 1	2,683 (84.7)	7,613 (78.2)		5,050 (82.9)	1,740 (78.5)	
	2+	485 (15.3)	2,127 (21.8)	<0.001	1,041 (17.1)	478 (21.6)	<0.001
Maternal non-manual social class	Non-manual	1,921 (60.6)	3,952 (47.5)		3,474 (57.0)	866 (49.7)	
	Manual	1,247 (39.4)	4,373 (52.5)	<0.001	2,617 (43.0)	877 (50.3)	<0.001
Maternal education = degree	CSE/vocational/o-level/a-level	2,529 (79.8)	7,501 (88.7)		5,083 (83.5)	1,567 (85.8)	
	Degree	639 (20.2)	960 (11.4)	<0.001	1,008 (16.6)	260 (14.2)	0.018
Type of dwelling = detached	Detached	654 (20.6)	1,238 (15.7)		1,168 (19.2)	258 (15.0)	
	Semi-detached/terrace	2,237 (70.6)	5,457 (69.0)		4,360 (71.6)	1,199 (69.5)	
	Flat	277 (8.7)	1,218 (15.4)	<0.001	563 (9.2)	268 (15.5)	<0.001
Maternal EPDS ≥12 in pregnancy	No	2,767 (88.8)	6,361 (82.8)		5,230 (87.5)	1,133 (81.7)	
	Yes	350 (11.2)	1,324 (17.2)	<0.001	748 (12.5)	254 (18.3)	<0.001

© 2018 Rai D, et al. *JAMA Psychiatry*

Maternal EPDS ≥12 postnatally	No	2,814 (88.8)	6,246 (81.7)		5,345 (87.8)	1,257 (79.0)	
	Yes	354 (11.2)	1,402 (18.3)	<0.001	746 (12.3)	335 (21.0)	<0.001
Major financial problems since pregnancy	No	2,854 (90.1)	7,567 (86.8)		5,492 (90.2)	1,439 (86.5)	
	Yes	314 (9.9)	1,149 (13.2)	<0.001	599 (9.8)	224 (13.5)	<0.001
		**Mean (SD)**	**Mean (SD)**	**P-value two-sided T-test**	**Mean (SD)**	**Mean (SD)**	**P-value two-sided T-test**
Maternal age at delivery		28.8 (4.4)	26.8 (5.0)	<0.001	28.5 (4.4)	27.2 (5.1)	<0.001
Maternal Crown-Crisp antenatal anxiety		4.5 (3.4)	5.1 (3.6)	<0.001	4.6 (3.4)	5.2 (3.6)	<0.001
Maternal Crown-Crisp postnatal anxiety		3.2 (3.1)	3.5 (3.4)	<0.001	3.2 (3.2)	3.6 (3.5)	<0.001

© 2018 Rai D, et al. *JAMA Psychiatry*

eTable 5. Risk of a Depression Diagnosis at Age 18 Years Among Children With Autism or Autistic Traits

		Outcome is diagnosed depression at age 18					
		Crude estimates			Adjusted estimates [a]		
**Exposure is:**	N [b]	RR [c]	(95% CI) [d]	p	RR [c]	(95% CI) [d]	p
ASD	3,168	0.41	(0.06 – 2.83)	0.36	0.50	(0.08 – 3.38)	0.48
Social communication impairments	2,830	1.52	(1.00 – 2.31)	0.052	1.60	(1.05 – 2.46)	0.029
Reduced speech coherence	2,841	0.76	(0.43 – 1.35)	0.35	0.77	(0.44 – 1.35)	0.37
Repetitive behaviours	2,857	1.43	(0.90 – 2.27)	0.13	1.41	(0.89 – 2.23)	0.15
Reduced sociability temperament	3,001	0.85	(0.55 – 1.32)	0.47	0.91	(0.59 – 1.41)	0.69

Notes: (a) Estimates were adjusted for child sex, parity, maternal age at delivery, maternal Crown-Crisp antenatal and postnatal anxiety, maternal EPDS antenatal and postnatal depression, maternal social class, maternal education, type of dwelling and the experience of major financial difficulties following conception of the study child. **(b)** Number of observations with complete data on exposure, covariates and diagnosis of depression at age 18. **(c)** Relative risk estimates were calculated using modified Poisson regression. **(d)** 95% confidence interval.

© 2018 Rai D, et al. *JAMA Psychiatry*

eTable 6. Risk of Adulthood Depression Among Children With Autism or Autistic Traits (Missing Covariate and Outcome Data Predicted With Multiple Imputation)

		Outcome is diagnosed depression at age 18					
		Crude estimates			Adjusted estimates [a]		
**Exposure is:**	**N [b]**	**RR [c]**	**(95% CI) [d]**	**p**	**RR [c]**	**(95% CI) [d]**	**p**
ASD	14,661	0.61	(0.17 – 2.17)	0.45	0.80	(0.23 – 2.81)	0.72
SCDC	8,110	**1.52**	**(1.09 – 2.213)**	0.015	**1.62**	**(1.16 – 2.28)**	0.005
Coherence	8,108	0.98	(0.67 – 1.43)	0.91	1.00	(0.69 – 1.46)	0.98
Repetitive behaviours	8,571	1.44	(1.00 – 2.09)	0.052	1.46	(1.00 – 2.12)	0.050
Sociability	10,042	0.79	(0.56 – 1.14)	0.21	0.83	(0.58 – 1.19)	0.31

Notes: (a) Estimates were adjusted for child sex, parity, maternal age at delivery, maternal Crown-Crisp antenatal and postnatal anxiety, maternal EPDS antenatal and postnatal depression, maternal social class, maternal education, type of dwelling and the experience of major financial difficulties following conception of the study child. **(b)** Number of observations with complete on exposure, with imputed data for covariates and diagnosis of depression at age 18. **(c)** Relative risk estimate were calculated using modified Poisson regression. **(d)** 95% confidence interval.

© 2018 Rai D, et al. *JAMA Psychiatry*

eTable 7. Association Between SCDC at Age 7 and a Depression Diagnosis at Age 18,[1] Mediated by the Experience of Being Bullied in Late Childhood/Early Adolescence[2] (Missing Covariate and Outcome Data Predicted With Multiple Imputation)

		Unadjusted association			Adjusted association [3]		
**Model fit statistics:**	Number of observations=	4,213			4,213		
	RMSEA=	0.051			0.043		
	CFI=	0.922			0.907		
	TLI=	0.898			.0880		
**Structural parameter estimates:**		B [4]	S.E. [5]	p [6]	B [4]	S.E. [5]	p [6]
Effect of exposure on mediator		0.208	0.040	<0.001	0.203	0.041	<0.001
Effect of mediator on outcome		0.430	0.067	<0.001	0.431	0.065	<0.001
Effect of exposure on outcome		0.142	0.070	0.043	0.189	0.073	0.009
Indirect effect		0.089	0.020	<0.001	0.087	0.020	<0.001
Total effect		0.231	0.070	0.001	0.276	0.072	<0.001
Proportion of total effect mediated		39%			32%		

Notes: (1) Depression was captured as a latent construct by means of four continuous measures of fatigue, concentration, sleep symptom score and depressive symptoms. (2) The experience of being bullied was captured as a latent construct by means of six binary scores capturing the child's status as a relational or overt victim at ages 8, 10 and 13. (3) Exposure-mediator and mediator-outcome associations were adjusted for offspring sex, maternal antenatal anxiety, maternal education and type of dwelling. (4) B = the unstandardised regression coefficient. (5) S.E. = standard error. (6) p = p-value.

© 2018 Rai D, et al. *JAMA Psychiatry*

eFigure 1. Flow Chart for Analysis Plan

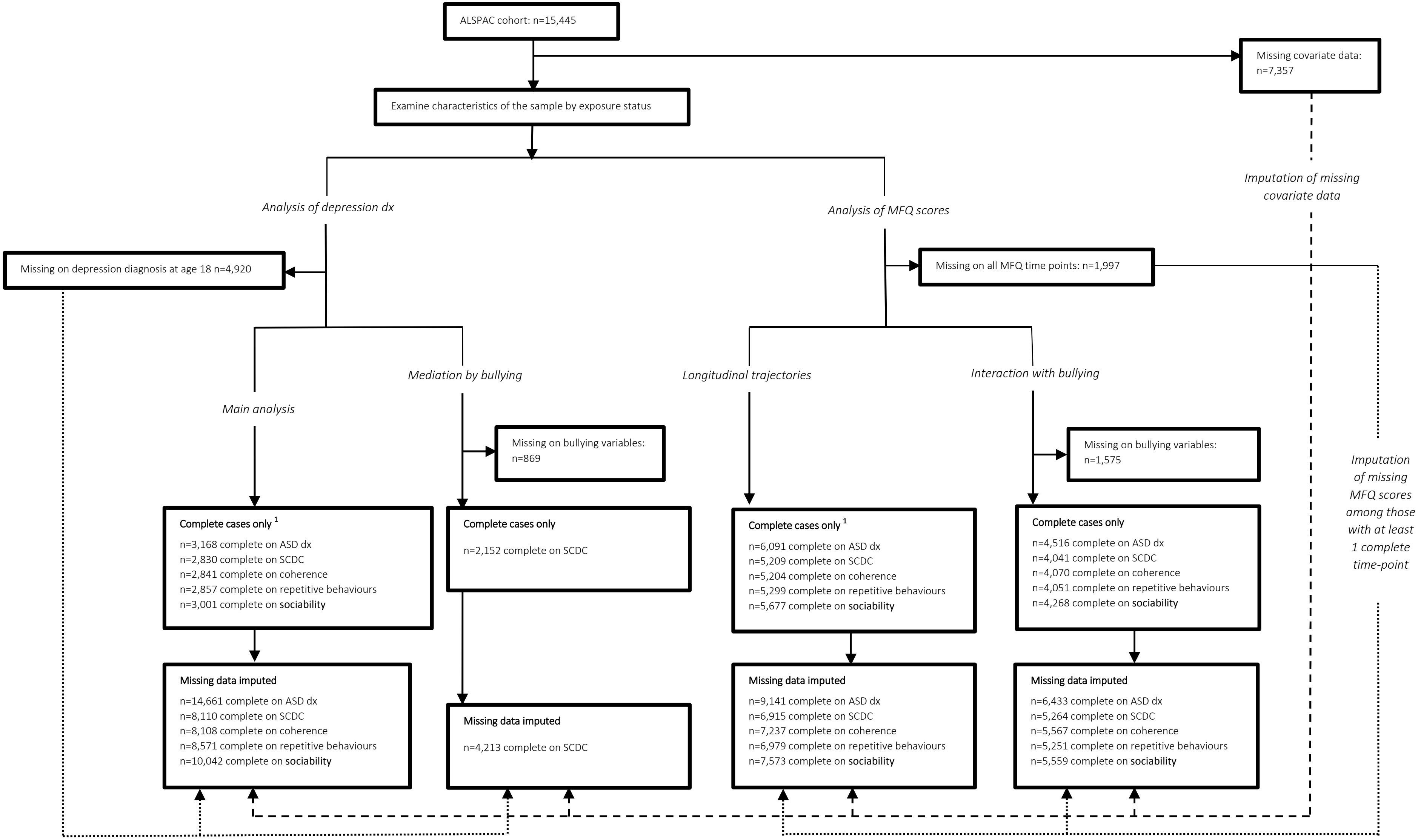

© 2018 Rai D, et al. *JAMA Psychiatry*

Notes: (1) We examined genetic confounding by adjusting for ASD polygenic risk among the subset of children who had been genotyped in supplementary analyses. (2) Mediation by bulling in late childhood and early adolescence was examined for autistic traits that were associated with diagnosed depression at age 18 in main analyses, i.e. SCDC and repetitive behaviours.

© 2018 Rai D, et al. *JAMA Psychiatry*

eFigure 2. Associations Between Continuous Polygenic Risk Score (PRS) for Autism and Autism Diagnosis and Autistic Traits

**Among those with or without diagnosis of autism** [b]	**Among those with a score in the worst decile or in the remaining 90 percentiles** [a] **of:**			
	**SCDC** [c]	**Coherence** [d]	**Repetitive Behaviours** [e]	**Sociability** [f]

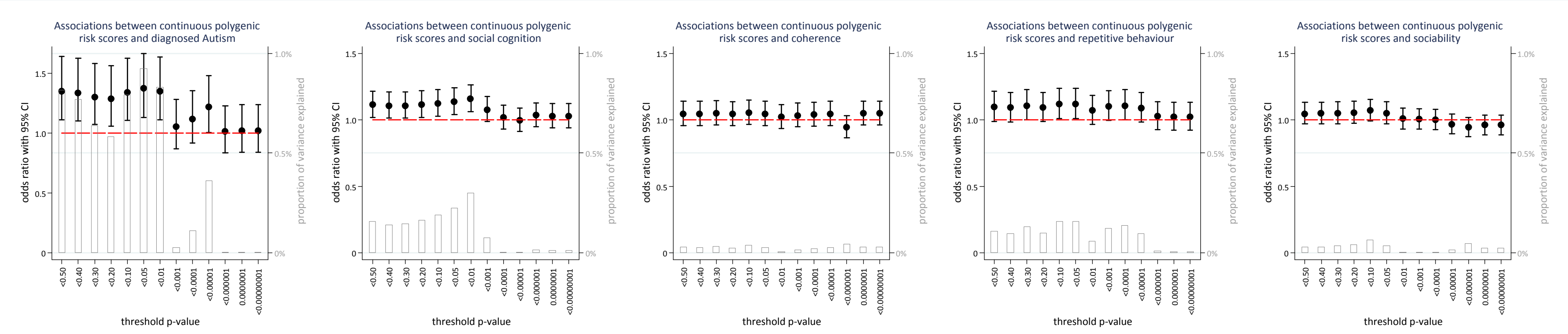

Notes: (a) Autistic traits were defined as membership to (approximately) the worst decile of autistic trait score distributions. **(b)** Estimates based on n=8,126 observations with complete data on PRS scores and autism diagnosis. **(c)** Estimates based on n=5,610 observations with complete data on PRS and SCDC scores. **(d)** Estimates based on n=5,722 observations with complete data on PRS and coherence scores. **(e)** Estimates based on n=5,718 observations with complete data on PRS and repetitive behaviour scores. **(f)** Estimates based on n=6,364 observations with complete data on PRS and sociability scores. Threshold p-values refer to the level of statistical significance at which single-nucleotide polymorphisms (SNPs) were identified in the discovery sample.

© 2018 Rai D, et al. *JAMA Psychiatry*

eFigure 3. Associations Between Dichotomized Measure of the Autism PRS (Top Decile Versus Bottom 90 Percentiles) and Autism Diagnosis and Autistic Traits

**Among those with or without diagnosis of autism [b]**	**Among those with a score in the worst decile or in the remaining 90 percentiles [a] of:**			
	**SCDC [c]**	**Coherence [d]**	**Repetitive Behaviours [e]**	**Sociability [f]**

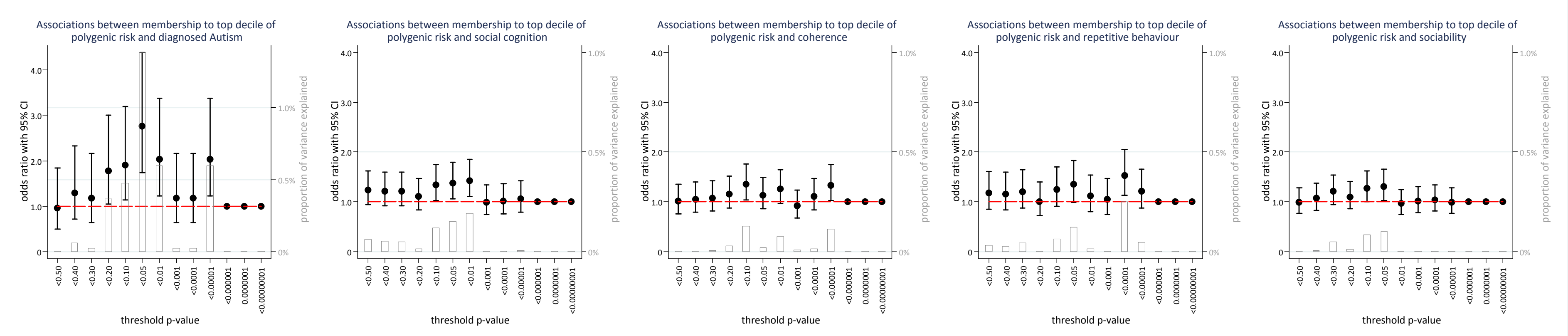

Notes: (a) Autistic traits were defined as membership to (approximately) the worst decile of autistic trait score distributions. **(b)** Estimates based on n=8,126 observations with complete data on PRS scores and autism diagnosis. **(c)** Estimates based on n=5,610 observations with complete data on PRS and SCDC scores. **(d)** Estimates based on n=5,722 observations with complete data on PRS and coherence scores. **(e)** Estimates based on n=5,718 observations with complete data on PRS and repetitive behaviour scores. **(f)** Estimates based on n=6,364 observations with complete data on PRS and sociability scores. Threshold p-values refer to the level of statistical significance at which single-nucleotide polymorphisms (SNPs) were identified in the discovery sample.

© 2018 Rai D, et al. *JAMA Psychiatry*

eFigure 4. Associations Between Continuous Polygenic Risk Score (PRS) for Autism and Diagnosed Depression at Age 18 Years (n=3,378)

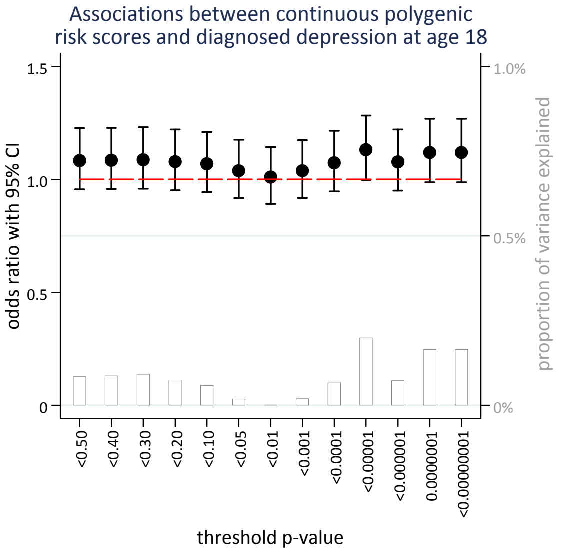

© 2018 Rai D, et al. *JAMA Psychiatry*

eFigure 5. Associations Between Continuous Polygenic Risk Score (PRS) for Autism and Bullying/Victimization at Any Time Point During Adolescence (n=5,032)

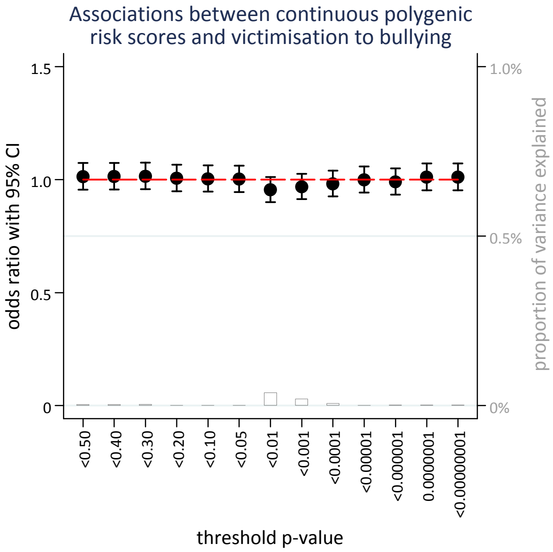

© 2018 Rai D, et al. *JAMA Psychiatry*

eFigure 6. Confounder-Adjusted Mean MFQ Scores Between Ages 10 and 18[a] (Missing Covariate and Outcome Data Predicted With Multiple Imputation)

Among those with (dashed) or without (solid) [a] diagnosis of autism [b]

Among those with a score in the worst decile (dashed) or in the remaining 90 percentiles (solid) of:

SCDC [c] | **Coherence [d]** | **Repetitive Behaviours [e]** | **Sociability [f]**

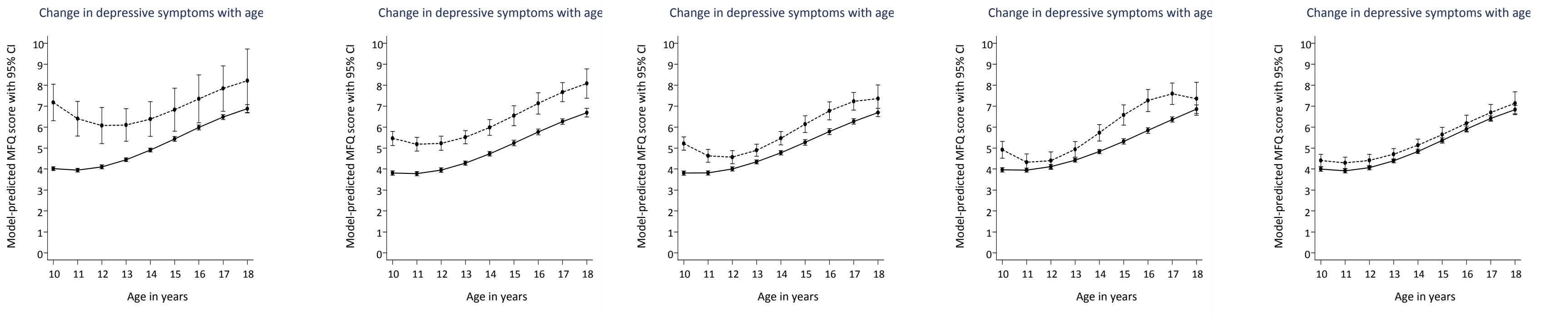

Notes: **(a)** Fitted means were calculated using xtmixed multilevel regression models with linear, quadratic and cubic terms for time. Trajectories were adjusted for child sex, parity, maternal age at delivery, maternal Crown-Crisp antenatal and postnatal anxiety, maternal EPDS antenatal and postnatal depression, maternal social class, maternal education, type of dwelling and the experience of major financial difficulties following conception of the study child. **(b)** Estimates based on n=9,141 observations with complete data on autism diagnosis and imputed data for covariates. **(c)** Estimates based on n=6,915 observations with complete data on SCDC scores and imputed data for covariates. **(d)** Estimates based on n=7,237 observations with complete data on coherence scores and covariates. **(e)** Estimates based on n=6,979 observations with complete data on repetitive behaviour scores and covariates. **(f)** Estimates based on n=7,573 observations with complete data on sociability scores and covariates.

© 2018 Rai D, et al. *JAMA Psychiatry*

eFigure 7. Confounder-Adjusted Mean MFQ Scores Among Children With/Without Autism or Autistic Traits and Exposed/Unexposed to Bullying[a] (Missing Covariate and Outcome Data Predicted With Multiple Imputation)

With or without a diagnosis of autism [b]

With or without a score in the worst decile of:

SCDC [c] | **Coherence** [d] | **Repetitive Behaviours** [e] | **Sociability** [f]

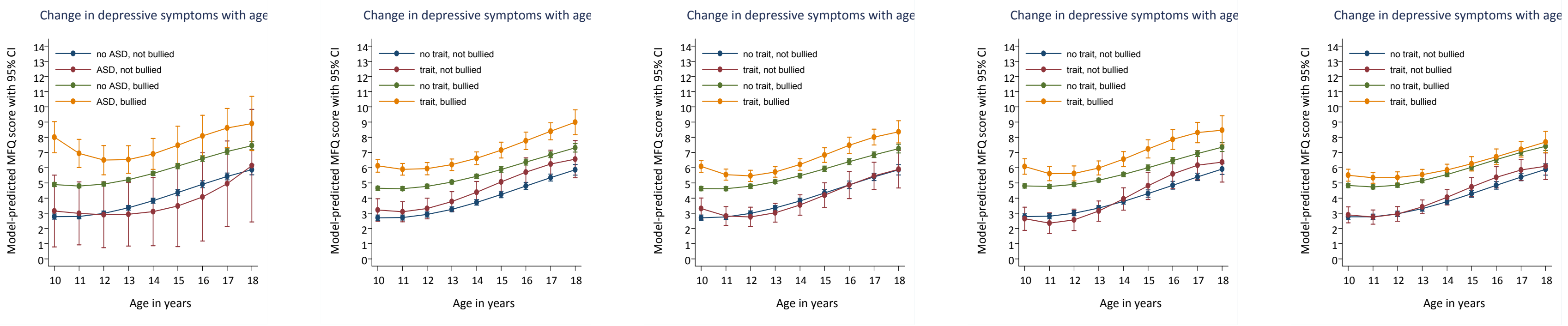

Notes: **(a)** Fitted means were calculated using xtmixed multilevel regression models with linear, quadratic and cubic terms for time. Trajectories were adjusted for child sex, parity, maternal age at delivery, maternal Crown-Crisp antenatal and postnatal anxiety, maternal EPDS antenatal and postnatal depression, maternal social class, maternal education, type of dwelling and the experience of major financial difficulties following conception of the study child. **(b)** Estimates based on n=6,433 observations with complete data on autism diagnosis and bullying variables, with imputed data for covariates. **(c)** Estimates based on n=5,264 observations with complete data on SCDC scores and bullying variables, with imputed data for covariates. **(d)** Estimates based on n=5,567 observations with complete data on coherence scores and bullying variables, with imputed data for covariates. **(e)** Estimates based on n=5,251 observations with complete data on repetitive behaviour scores and bullying variables, with imputed data for covariates. **(f)** Estimates based on n=5,559 observations with complete data on sociability scores and bullying variables, with imputed data for covariates.

© 2018 Rai D, et al. *JAMA Psychiatry*
